# Enhancing Nutrition Care Through Real-Time, Sensor-Based Capture of Eating Occasions: A Scoping Review

**DOI:** 10.3389/fnut.2022.852984

**Published:** 2022-05-02

**Authors:** Leanne Wang, Margaret Allman-Farinelli, Jiue-An Yang, Jennifer C. Taylor, Luke Gemming, Eric Hekler, Anna Rangan

**Affiliations:** ^1^Charles Perkins Centre, Faculty of Medicine and Health, University of Sydney, Sydney, NSW, Australia; ^2^Department of Population Sciences, Beckman Research Institute, City of Hope, Duarte, CA, United States; ^3^The Design Lab, University of California, San Diego, San Diego, CA, United States; ^4^Herbert Wertheim School of Public Health and Human Longevity Science, University of California, San Diego, San Diego, CA, United States

**Keywords:** wearable sensors, dietary assessment, dietary intake, food timing, food intake detection, nutrition care, sensors, scoping review

## Abstract

As food intake patterns become less structured, different methods of dietary assessment may be required to capture frequently omitted snacks, smaller meals, and the time of day when they are consumed. Incorporating sensors that passively and objectively detect eating behavior may assist in capturing these eating occasions into dietary assessment methods. The aim of this study was to identify and collate sensor-based technologies that are feasible for dietitians to use to assist with performing dietary assessments in real-world practice settings. A scoping review was conducted using the PRISMA extension for scoping reviews (PRISMA-ScR) framework. Studies were included if they were published between January 2016 and December 2021 and evaluated the performance of sensor-based devices for identifying and recording the time of food intake. Devices from included studies were further evaluated against a set of feasibility criteria to determine whether they could potentially be used to assist dietitians in conducting dietary assessments. The feasibility criteria were, in brief, consisting of an accuracy ≥80%; tested in settings where subjects were free to choose their own foods and activities; social acceptability and comfort; a long battery life; and a relatively rapid detection of an eating episode. Fifty-four studies describing 53 unique devices and 4 device combinations worn on the wrist (*n* = 18), head (*n* = 16), neck (*n* = 9), and other locations (*n* = 14) were included. Whilst none of the devices strictly met all feasibility criteria currently, continuous refinement and testing of device software and hardware are likely given the rapidly changing nature of this emerging field. The main reasons devices failed to meet the feasibility criteria were: an insufficient or lack of reporting on battery life (91%), the use of a limited number of foods and behaviors to evaluate device performance (63%), and the device being socially unacceptable or uncomfortable to wear for long durations (46%). Until sensor-based dietary assessment tools have been designed into more inconspicuous prototypes and are able to detect most food and beverage consumption throughout the day, their use will not be feasible for dietitians in practice settings.

## Introduction

In the past decade, there has been a shift away from traditional eating patterns (1). The proportion of adults moving away from the conventional three main meals per day toward smaller but more frequent meals and snacks is increasing (1). These dietary trends are more apparent in young adults whose food intake pattern is less structured (2) and more spontaneous (3). Thus, there are implications for self-reported dietary intakes as snacking occasions are frequently omitted when using traditional dietary assessment methods (4) due to users forgetting or declining to self-report (5). There have been technological advances in the collection and processing of dietary information, with web- and mobile application (app)-based food diaries and computerized adaptations of the 24-h recall such as the National Cancer Institute's Automated Self-Administered 24-h recall (ASA24) (6, 7) and the Measure Your Food on One Day 24-h Recall (myfood24) (8) becoming the norm. However, digital food diaries still rely on user-initiation and self-report (9) and the computerized 24-h recalls have similar limitations to that of traditional dietary assessment methods and have been documented to omit food items (10, 11).

Newer technologies utilizing wearable and non-wearable sensors offer an objective and passive means to measure behaviors related to food and beverage consumption. They provide convenience for users by removing the burden of self-report (12) and may be of higher measurement accuracy, thereby presenting advantages over traditional dietary assessment methods. The capabilities of these technologies are constantly evolving and improving given the relatively new nature of this field (13) and have the potential to advance the Nutrition Care Process (NCP) (14), a framework that guides the Medical Nutrition Therapy (MNT) dietitians provide.

Nutrition Assessment is the first step of the NCP, and it involves the collection and documentation of information, including food-related history (14). In addition to determining what foods are eaten during meals and less structured eating occasions, dietitians may examine how foods are eaten. This may be enhanced *via* the use of sensors as their ability to detect hand-to-mouth (HTM) actions and biting, chewing, and swallowing of food enables them to provide valuable information about how someone eats, including the timing of consumption, duration of eating episodes, and frequency of eating occasions. Sensors and wearables such as wearable cameras that take images at frequent and regular intervals throughout the day (e.g., every 30 s) (15) may be used to reveal what people are eating and combinations of sensors can be used to determine food type and calorie intake when chewing motions are detected (16). Furthermore, real-time detection of eating gestures allows for the delivery of timely prompts that remind users to record their dietary intake, reducing the chances of inaccurate dietary recalls as a result of memory decay (5). This is a form of event-contingent Ecological Momentary Diet Assessment (EMDA) (17). Beyond their value in rigorous measurement, the use of prompts in tandem with sensing technologies can assess a person's psychological and physical state and social and food environmental surroundings when eating occasions are detected, helping a dietitian understand why a client eats as they do.

The remaining steps of the NCP are Nutrition Diagnosis, Intervention, and Monitoring/Evaluation (14), all of which could also be enhanced with the use of sensor-based devices. Nutrition Diagnosis involves the naming of a specific nutrition problem based on data collected during nutrition assessment. Sensors may be able to detect the signs and symptoms of nutrition problems such as excessive or inadequate energy intake based on the correlation between energy intake and HTM motions (18); swallowing or chewing difficulties using devices that measure these gestures to detect eating (19); and irregular eating patterns based on the detected start time of eating occasions and the consistency of these times across multiple days. This type of information collected by the sensor-based devices may assist dietitians in establishing a nutrition diagnosis more efficiently. During Nutrition Intervention, the dietitian selects an appropriate intervention to be directed toward the root cause of the nutrition problem (14). Again, with the use of sensor-based devices, timely prompts that remind users of this intervention, may be delivered upon the detection of an eating event (20) to enable clients to achieve their dietary goals. This can be in the form of a short message that appears on a smartwatch or a connected smartphone that informs the user of an action or food choice that can be made to motivate them toward achieving their dietary goal. During the Monitoring/Evaluation stage, users' dietary habits in terms of the rate of eating (e.g., bites/chews per minute) and dynamic pattern of food consumption (21, 22) may be monitored and evaluated remotely and in real-time between consultation sessions (23).

Although some authors from dietetic backgrounds have already integrated sensor-based devices into their studies (24, 25), specifically the wrist-worn device known as the Bite Counter that detects HTM motions, there is currently no systematic process in place to evaluate the feasibility of other devices for dietetic practice. For sensor-based devices to be useful to dietitians in real world practice, engineers building the devices need to be cognizant of the practicality of devices. The sensor will need to be discrete and comfortable to wear throughout the day so as not to inhibit client/patient compliance with using the device. Battery life will need to be sufficient to cover waking hours without recharging. Ideally, multiple functions could be incorporated into one device rather than requiring numerous sensors. The field of physical activity demonstrates how passive sensing can move from more obtrusive bulky accelerometers worn on the hip to watch-like devices worn on the wrist (26).

In an area that is rapidly evolving, a scoping review was selected as the most appropriate method to identify both peer-reviewed studies and gray literature between January 2016 and December 2021 that assessed the use of sensor-based devices in detecting eating and/or drinking occasions. These devices were then evaluated with a set of five feasibility criteria to identify and collate those that could potentially be useful for assisting dietitians in performing dietary assessments as part of the NCP in real-world practice settings.

## Materials and Methods

### Protocol and Registration

Our protocol was drafted using the Open Science Framework (OSF) Preregistration template and registered prospectively with OSF on 11/01/2022 (https://doi.org/10.17605/OSF.IO/EYH5A).

### Eligibility Criteria

The selection criteria informed the population, concept, and context relevant to the review's objective. This scoping search was inclusive of any scientific paper published between January 2016 and December 2021 that used sensor-based devices to passively detect and record the initiation of eating in real-time, as shown in Table 1. As this field is rapidly advancing and sensor-based technologies evaluated in earlier papers are likely to have been further developed, we focus on the literature from the past 5 years.

**Table 1:** Inclusion and exclusion criteria of the scoping review.

**Selection criteria**	**Inclusion criteria**	**Exclusion criteria**
Population	• Adult participants 18 years or older • Participants 17 years or younger if the device can potentially be used in adults	• Animal studies
Concept	• Device identified and recorded, or had the capacity to identify and record, the start time of an eating and/or drinking occasion over multiple days in real-time • Passive, sensor-based device that did not require user input • The most recent paper published about the device by a particular research group if it supersedes earlier papers	• Delay ≥20 mins between the detected gesture or proxy for eating or drinking and the true occasion • Instruments that measured usual, average or overall intake; did not identify and record the start time of eating and/or drinking; were used solely for administering dietary interventions; measured enteral or parenteral feeding; were displaced or discontinued at the time of the search; or required user input • Web-, mobile app-, or paper-based dietary assessment tools
Context	• Papers published between 2016 and 2021 inclusive and in English • Case studies, conference proceedings, dissertations, and opinion papers in both peer-reviewed and gray literature • No limitations on geographic location • Study could be conducted in laboratory, semi-controlled, or free-living settings. However, the device had to be feasible for use in free-living settings in terms of user acceptability and comfort. • Any study or review of studies that evaluated the performance of a device	• All papers published before 2016 and papers published in all other languages • Papers that did not evaluate the device's performance • Papers solely describing the data processing pipeline and algorithm of the model without describing the design of the device hardware

### Information Sources and Search

For the scoping review, seven databases were searched: ACM digital library, CINAHL (EBSCO), EMBASE (Ovid) (EMBASE, RRID:SCR_001650), IEEE Xplore (IEEE), PubMed, Scopus (Elsevier), and Web of Science (Clarivate Analytics). Sources of gray literature searched included: Google, Trove, MedNar, OpenGrey and official websites of international and government organizations. The search strategy was optimized to extract both peer-reviewed studies and gray literature and was developed and refined with the aid of an experienced librarian with expertise in database searches. The complete search strategy for Ovid MEDLINE can be found in the Appendix A in Supplementary Material. The final search results were exported into EndNote (EndNote, RRID:SCR_014001), and duplicates were removed.

### Screening and Selection of Evidence

The titles and abstracts of all studies were reviewed against the eligibility criteria. If the title and/or abstract mentioned a sensor-based device that detected eating, the study was included in the initial screening stage to be assessed in the full-text screening stage.

**Figure 1:**
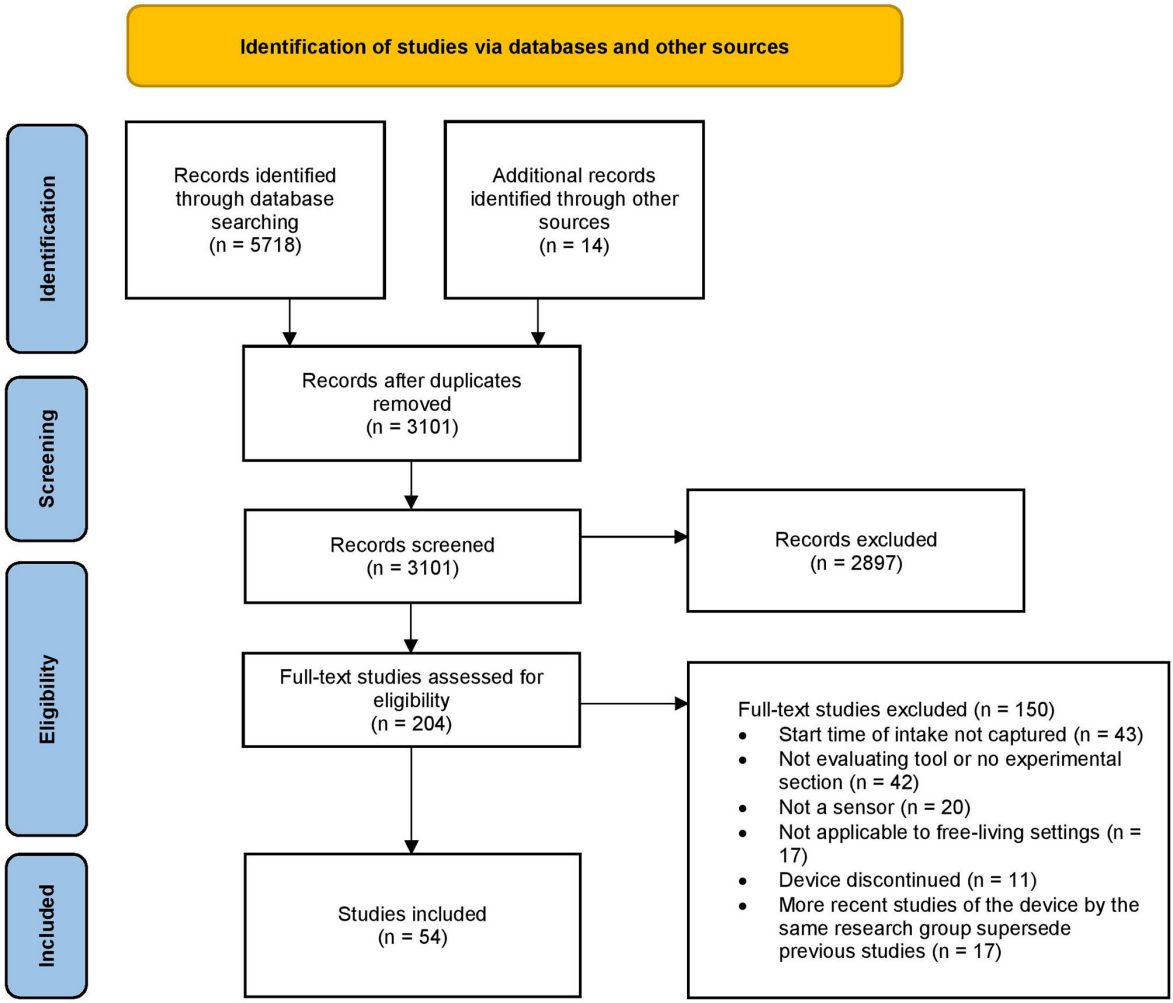
Flow chart of included studies.

Studies were further excluded during the full text screening stage if they did not evaluate device performance or if the same research group conducted a more recent study describing a device that superseded previous studies of the same device. Studies evaluating a device that did not have the capacity to detect and record the start time of food intake, did not use sensors, were not applicable for use in free-living settings, or were discontinued at the time of the search were also excluded (Figure 1).

### Data Charting Process and Data Items

The data extraction table was developed specifically for this review (Supplementary Table 1). In the table, the devices were categorized according to device placement. For each device, the following data items were included:

1. The type(s) of sensors used, and the number of each sensor included in the device.
2. The type(s) of intake (e.g., food, beverages) and eating proxy or gesture (e.g., HTM motions, chewing, swallowing, etc.) measured.
3. Ground truth method, evaluation metric(s), and performance of sensors.
4. Advantages and disadvantages of the study, including both the study design as well as the feasibility of the device for assisting with dietary assessment in real-world settings according to the five feasibility criteria described below.
5. Experiment details including the setting, duration, number of food and/or beverage types tested, and number of participants.
6. The data processing pipeline including the classification algorithm used, sampling rate, and the number of features. The number of papers using each type of classification algorithm was tabulated in a separate table (Supplementary Table 2). This table was developed by a dietitian (LW) and a geospatial data scientist (JY). A short description of each algorithm using plain language is provided.

### Synthesis of Results

Devices in the data extraction table were further evaluated against a set of five feasibility criteria to identify those that were suitable for assisting dietitians with performing dietary assessment (Step 1 of the NCP) in real-world settings (Table 2). The five feasibility criteria were: (i) the study's evaluation metric showing an average accuracy or F1-score of ≥80% in detecting individual eating proxies such as HTM movements, biting, chewing, or swallowing; eating bouts (periods of eating within an eating episode); or entire eating episodes (ii) in free-living, semi-free-living, or laboratory settings where participants were free to choose their own foods and activities. The feasibility cut-off score of ≥80% was selected based on the performance of the Bite Counter device employed in dietitian-led trials (24, 25). This device achieved an F1-score of 82% in free-living settings (81). (iii) The device had to be discreet, socially acceptable, and comfortable for the user to wear for long durations. This was determined based on user feedback where available and the appearance and description of the device hardware. Due to the emerging nature of this field, the design of the hardware did not need to match the quality and appearance of off-the-shelf wearables at this stage. However, devices that were large, obtrusive, and not disguisable as everyday accessories such as glasses were considered socially unacceptable. (iv) The battery life of the device on a single charge needed to exceed 12 h or one waking day and (v) the data processing pipeline and algorithm used needed to be able to classify a segment of data in real-time as either eating or not eating within 5 mins. Five minutes was chosen to capture all meals, including snacks.

**Table 2:** An overview of all identified devices compared with the five feasibility criteria used to determine suitability for use by dietitians in real-world settings.

**Reference**	**Accuracy^a^**	**Real-world applicability^b^**	**Social acceptability^c^**	**Battery life^d^**	**Real-time processing^e^**	**Both eating & drinking^f^**
**Wrist-Worn Devices (*****n*** **= 18)**						
Ortega Anderez et al. (27)	√	×	–	×	×	√
Stankoski et al. (28)	√	√	√	×	×	×
Solis et al. (29)	√	√	√	×	×	×
Lin and Hoover (30)	√	×	√	×	–	×
Sharma et al. (31)	×	√	√	×	×	×
Kyritsis et al. (32)	√	√	√	×	×	×
Gomes et al. (33)	×	√	×	×	–	√
Sen et al. (34)	√	–	√	√	√	×
Zhang et al. (35)	√	×	×	×	–	×
Gan et al. (36)	√	×	×	×	–	×
Fortuna et al. (37)	√	×	√	×	×	×
Zhang et al. (38)	√	×	√	×	–	×
Kim et al. (39)	√	×	√	×	–	×
Siddhartha Varma et al. (40)	√	×	×	×	–	√
Lee et al. (41)	√	×	√	×	–	×
Navarathna et al. (42)	×	√	√	×	–	×
Kamachi et al. (43)	×	×	√	×	×	×
Hnoohom et al. (44)	√	×	√	×	×	×
**TOTAL “√”**	14	6	13	1	1	3
**Neck-Worn Devices (*****n*** **= 9)**						
Shin et al. (45)	√	×	×	×	√	×
Bi et al. (46)	√	×	×	×	√	√
Hussain et al. (47)	√	×	×	×	√	√
Chun et al. (48)	×	√	√	√	×	×
Kalantarian et al. (49)^*^	√	×	√	×	–	√
Kalantarian et al. (49)^*^	×	×	×	×	–	√
Lee et al. (50)	×	×	×	×	–	√
Zhang et al. (51)	×	√	×	√	×	×
Nguyen et al. (52)	×	×	×	×	–	√
**TOTAL “√”**	4	2	2	2	4	6
**Ear-Worn Devices (*****n*** **= 9)**						
Kondo et al. (53)	√	×	√	×	×	×
Bi et al. (54)	√	√	×	√	√	×
Bi et al. (55)	√	×	√	×	–	×
Islam et al. (56)	√	×	√	×	–	×
Taniguchi 2018 (57)	√	×	√	×	√	×
Taniguchi et al. (58)	–	×	√	×	×	×
Blechert et al. (59)	√	√	√	×	×	×
Papapanagiotou et al. (60)	√	√	×	×	×	×
Bedri et al. (61)	√	√	×	×	√	×
**TOTAL “√”**	8	4	6	1	3	0
**Glasses Devices (*****n*** **= 7)**						
Farooq et al. (62)	√	√	×	×	×	√
Farooq et al. (63)	√	×	×	×	√	×
Zhang et al. (64)	√	√	–	×	√	×
Chung 2017 (65)	√	×	–	×	–	×
Ghosh et al. (66)	√	√	√	×	√	×
Bedri et al. (67)	√	√	×	√	√	√
Selamat et al. (68)	√	×	√	×	–	×
**TOTAL “√”**	7	4	2	1	4	2
**Other Devices (*****n*** **= 10)**						
Lin et al. (69)	√	×	–	×	–	×
Nyamukuru et al. (70)	√	×	–	×	√	×
Wang et al. (71)	√	×	×	×	–	×
Zhang et al. (72)	√	×	√	×	√	×
Chun et al. (73)	√	√	×	×	×	×
Lin et al. (30)	–	×	×	×	–	×
Gan et al. (36)	√	×	×	×	–	×
Chun et al. (74)	√	√	×	×	×	×
Yang et al. (75)	×	×	×	×	×	×
Chen et al. (76)	√	×	√	×	–	×
**TOTAL “√”**	8	2	2	0	2	0
**Multi-Position Devices (*****n*** **= 4)**						
Johnson et al. (77)	√	×	×	×	–	×
Hussain et al. (78)	√	×	√	×	–	√
Farooq et al. (79)	×	√	×	×	×	√
Mirtchouk et al. (80)	×	√	×	×	×	×
**TOTAL “√”**	2	2	1	0	0	2

a *An average accuracy or F1-score of ≥80% in detecting individual eating proxies, eating bouts, or entire eating episodes*.

b *Device testing occurred in free-living, semi-free-living, or laboratory settings where participants were free to choose their own foods and activities*.

c *A device that was discreet, socially acceptable, and comfortable for the user to wear for long durations determined by user feedback or the appearance and description of the device hardware*.

d *The study reported a battery life on a single charge of >12 h or one waking day*.

e *Sensor data were classified in real-time as either eating or not eating within 5 mins*.

f *Devices that could detect, but not necessarily distinguish between, eating and drinking events. All devices could detect eating events. This was not one of the five feasibility criteria*.

* *Two devices were evaluated by this study. The first was an air microphone and the second was a piezoelectric sensor. Both were worn on the neck*.

*“√” indicates that the criterion has been met; “ × ” indicates that the criterion has not been met; and ‘–' indicates that the criterion was not reported by the study and could not be inferred by the authors*.

Table 2 was formatted as a checklist, where each criterion occupied a column, and each device occupied a row. An additional column for the detection of beverages was also included. Like the data extraction table (Supplementary Table 1), devices were categorized by device location (wrist, neck, ear, glasses, other, multi-position).

## Results

### Study Selection

The search yielded 5,732 results in total: 5,718 studies were identified by searching scientific databases and another 14 were identified by searching the gray literature and the reference lists of identified eligible studies. After removal of duplicates, 3,101 abstracts were screened by title and abstract and then full text for eligibility (Figure 1). A total of 54 studies for sensors were identified in this review. Reasons for full-text exclusions included: (i) the devices not identifying or recording the start of food and/or beverage intake time, (ii) the study not having an experimental section or only evaluating the data-processing pipeline without describing the hardware, (iii) the study not describing a sensor-based device, (iv) the device not being applicable to free-living settings, (v) the study describing a discontinued device, and (vi) the study being superseded by a more recent paper of the same device by the same research.

### Study Characteristics

Fifty-three unique devices and four combinations of devices (77–80) identified from 54 studies were included in the data extraction table (Supplementary Table 1). Of the 53 unique devices, 18 devices were placed on the wrist (27–44); nine were worn around the neck (45–52); nine were placed in or around the ears (53–61); seven were glasses-like devices (62–68); and ten were categorized as “Other Devices” (30, 36, 69–76) where devices were worn in less common locations of the body or were non-wearable (69, 72).

Most of the devices were trained and/or tested in laboratory settings (*n* = 32). This was followed by devices trained and/or tested in free-living settings (*n* = 22). Two of the devices were trained and tested in both free-living and laboratory settings for different food types (58, 74) and one was tested in semi-free-living settings (60).

Most of the devices (*n* = 47) were assessed using one or more validation methods as the “ground truth” for comparison with inferred eating and/or drinking occasions. The ground truth was captured by objective methods such as video or image recording, researcher annotation or observation, and electromyography (EMG) waves and voltages (*n* = 23); subjective methods such as user annotation of recordings, human memory and self-report, activity logging, and the use of a push button to mark chewing or swallowing events (*n* = 21); or both (*n* = 3).

### Results by Device Type

A summary of the devices' performance, including the advantages and disadvantages of each device category as well as the study design used to evaluate the device(s) are described below. Further details are available in Supplementary Table 1.

#### Wrist-Worn Devices

Devices worn on the wrist measured intake *via* the HTM movement. Therefore, a major advantage of wrist devices is the potential for it to detect both eating and drinking as it does not rely on chewing or swallowing activity. Both food and beverage intake were able to be identified in 5/18 wrist devices (27, 33, 37, 40, 44) and three of these could also distinguish between eating and drinking movements (27, 33, 40). The algorithms used by the wrist devices to detect HTM movements could be adapted to different handedness depending on the individual. Most were worn on the dominant wrist whilst one was worn on the utensil-operating hand (32). Wrist devices are assumed to have a high user acceptability especially with the use of commercial smartwatches (28, 29, 32, 42) or models that are compatible with popular commercial health tracking devices (37). However, user acceptability was not formally tested for most wrist devices. Whilst none of the devices could provide an assessment of the actual foods consumed or the energy and nutrient intake, one device could distinguish between the use of different types of cutleries (28), one could distinguish between a limited number of food types (ramen, pasta, bread, onigiri, gyudon, cake) (43), and one could capture (but not provide an assessment of) food type *via* an embedded camera (34).

The main disadvantages of the studies that used wrist devices included: generating false positives for hand actions close to the face (33, 42), and using a limited number of foods, utensils, and/or behaviors to train and test the model, leading to overly optimistic results (27, 30, 34, 36, 38); and users being required to wear the wrist device on both wrists (35).

#### Neck-Worn Devices

Devices placed on the neck used a variety of sensors such as microphones, piezoelectric sensors, and proximity sensors to identify chewing and/or swallowing activity. Unlike microphones, the use of piezoelectric sensors (pressure sensor) does not generate privacy concerns (47, 49, 52). Most sensors used in neck-worn devices captured both chewing and swallowing signals and were therefore capable of differentiating between food and liquid intake based on the pattern of chewing and swallowing (46, 47, 49, 50). These four devices could also differentiate between a limited number of food types ranging from three to 17 individual foods.

Like wrist-worn devices, a drawback of the studies that described devices worn on the neck was the strict experimental protocols that did not reflect real-world eating scenarios. This included experiments being conducted in settings with low environmental noise (46, 49) and using a limited number of foods or behaviors (46, 47, 49). Reduced performance was seen in neck devices for users with a higher (51) or lower body mass index (BMI) (46) and for users with dysphagia (50). Both tight-fitting and loose-fitting neck sensors scored modestly for user comfort (47, 50) and it was also common for loose-fitting devices to move out of place during physical activity (45, 48). None of the neck devices could provide an assessment of energy and nutrient intake.

#### Ear-Worn Devices

Similar to wrist-worn devices, a major strength of using devices in and/or around the ears is the potential user acceptability of an in-ear earphone device (56, 58) or two discrete electrodes behind the ear (59). Seven of the nine ear-worn devices used chewing as a proxy for eating (54, 55, 57–61), two of which deliberately used soft foods such as yogurt, ice cream, puree, and custard to train the devices' algorithms as they are more prone to being missed (55, 60). However, it was unclear how the devices performed in detecting these specific foods as the authors did not report on these foods separately.

Despite being unobtrusive, subjects reported that they would only wear the earbud-like device continuously for 4 h due to discomfort (60). Other ear-worn devices were still in preliminary stages, testing only two food types (57, 58) and requiring manual adjustments by researchers before consuming each food type (57). Some devices were only worn during mealtimes, so it was difficult to determine whether these algorithms were able to classify eating from other activities (53, 58). Studies that used chewing as a proxy for eating saw a lower performance in subjects with bruxism and/or nail biting (59). Movements such as walking (54), talking (56), and strong head movements (59) were commonly confused with chewing. None of these devices were evaluated for drinking detection, could identify or capture food type, or were able to provide an assessment of energy and nutrient intake.

#### Glasses-Like Devices

Like most ear-worn devices, glasses-like devices also used chewing activity to detect eating. However, rather than detecting chewing *via* jaw motions, it was detected *via* temporalis muscle activation located on the side of the forehead. Despite this, one device was still able to detect drinking where the duration between sips was <30 secs (67), although it was not capable of differentiating between eating and drinking occasions. Two devices were designed as a pair of glasses embedded with a small camera and were able to capture but not classify food type (66, 67). One of them could detect chewing within 1.1002 milliseconds (66). Another device with EMG sensors (64) was able to detect eating start time with an error of 24.8 secs. The sensor(s) used in three devices (62, 63, 66) were detachable from the device and could be mounted onto any off-the-shelf glasses, increasing user acceptability in those who regularly wear glasses.

All glasses-like devices identified in the scoping review required the frame of the glasses to be in contact with the temporalis muscle to measure chewing. This meant that the glasses had to be tightly fitted and could be affected by perspiration or hairs between the skin and the glasses. Thus, users who do not wear glasses regularly may experience discomfort (68). Subjects also raised privacy concerns regarding the continuous capture of the cameras attached to the glasses (67). Other disadvantages associated with using chewing activity to identify eating occasions included having a lower performance when detecting soft foods. None of the devices in this category could provide an assessment of energy and nutrient intake.

#### Other Devices

Devices that were categorized under “other” included wearable (*n* = 8) and non-wearable apparatuses (*n* = 2). Non-wearable technology included a WiFi transmitter and a receiver, using interferences in the WiFi signaling between a WiFi access point and a smartphone to deduce human body motion (69). Another non-wearable was a smart fork that detected food pick-up gestures and bite quantity using a load cell embedded inside the fork to measure the weight of each bite (72). This could potentially be used to estimate caloric intake. Other wearable devices included a headband (70, 71), a pin (76), a sensor placed on the chin (73), on the finger (30, 36), on the lower teeth (74), and a hip-worn Global Positioning System (GPS) and accelerometer (75).

Most of the devices in this category were still in preliminary developmental stages and were tested on a limited number of foods and/or behaviors in laboratory settings (30, 36, 68–72). However, devices worn on the chin (73) and teeth (74) were small and discreet and had been tested in free-living settings. One device was able to broadly categorize food by type (moist/dry and hot/cold) using changes in intraoral temperature and jawbone movement (74). Another device that included an accelerometer and GPS worn on the hip (75) was able to predict food purchasing and eating events up to 4 mins ahead of time with 74 and 73% mean accuracy respectively, even in in-home settings. This was the only device that could detect eating prior to the event which is ideal for providing just-in-time dietary feedback or reminders to guide users toward their dietary and health goals. None of the devices in this category were evaluated for drinking detection.

#### Multi-Position Devices

Devices in this category involved sensors placed on multiple locations of the body. Using a combination of devices has the advantage of being able to counteract each other's drawbacks (79, 80). The study by Mirtchouk and Kleinberg (80) used one earbud and two smartwatches worn on both wrists. Whilst the earbud only detected chewing sounds associated with eating, the smartwatch detected HTM movements for both eating and drinking occasions and also mitigated any false positives that may be picked up by the microphone on the earbud. The sounds picked up by the earbud can also help to reduce false positives generated by the smartwatches when the user performs other hand gestures close to the face. Two devices in this category were able to distinguish between eating and drinking events (78, 79), with one also able to distinguish between six food types, including water (78).

A major drawback of using multiple devices would be user comfort and acceptability, especially when worn for long durations in social settings. Only one device combination, a wrist and neck sensor, evaluated user experience (78) and reported that most participants were comfortable with wearing the two instruments, especially the wrist sensor (smartwatch). This study (78), as well as one other (77), was conducted in laboratory settings with a limited number of activities, subjects, and food types. None of the devices could provide an assessment of energy and nutrient intake.

### Summary of Devices Most Promising for Dietetic Practice

Based on the five feasibility criteria, none of the devices identified in this scoping review were deemed feasible for use by dietitians to assist with conducting dietary assessments. The feasibility criteria were, in brief, consisting of an accuracy ≥80%; tested in settings where subjects were free to choose their own foods and activities; social acceptability and comfort; a long battery life; and a relatively rapid detection of an eating episode.

The most common reason for exclusion was an insufficient or lack of reporting on battery life, accounting for 91% of the devices. The wrist-worn category had the highest proportion of devices that did not meet this criterion. This was followed by the use of a limited number of foods and/or behaviors to test device models (63%), where devices from the “other” category had the highest proportion not meeting the criterion. Forty-six percent of devices predominately from the neck-worn category were socially unacceptable or uncomfortable to wear for long durations. Thirty-five percent of devices had not been tested or calculated for real-time applicability. This was mostly seen in the multi-position device category. Twenty-one percent of devices did not meet the accuracy or F1-score of ≥80% criterion. This was also predominately contributed by the neck-worn devices. Note that the percentages do not add up to 100% as studies could have had more than one reason for exclusion. Twenty-three percent of devices, predominately from the neck-worn category, were evaluated for the detection of behaviors related to both eating and drinking. Table 2 provides an overview of the 57 devices and device combinations and the feasibility criteria they fulfilled and did not fulfill.

Select features of devices that fulfilled four out of the five feasibility criteria (34, 54, 66, 67) can be used to highlight their potential application in dietitian-led MNT. These devices included:

A pair of glasses embedded with gyroscope, accelerometer, and proximity sensors (67) (feasibility criterion iii unfulfilled); a wrist-worn device (34) (feasibility criterion ii unfulfilled), a pair of off-the-shelf eyeglasses with an attached sensor system (accelerometer) (66) (feasibility criterion iv unfulfilled), and a headband that housed a contact microphone beside the ear (54) (feasibility criterion iii unfulfilled).

Dietitian-led MNT is guided by the four step NCP. During Nutrition Assessment (Step 1), dietitians will usually conduct a diet history to estimate nutritional adequacy and meal patterns but will sometimes ask patients to keep a diet record in advance of their consultation. However, patients sometimes miss meals and snacks or forget to record in paper- or app-based dietary records (82). The headband device detects eating passively using chewing sounds, with a 3-mins delay between the start of eating and eating detection (54). This type of device may be used to remind users to document food intake retrospectively in the form of an event-triggered EMDA. This may reduce the burden of self-initiation and reduces the number of missed occasions resulting from when users forget to report. The wrist-worn and two glasses-like devices have a relatively quick processing time (60 sec or less) and can be used to passively detect and capture the types of foods and beverages consumed *via* the embedded camera. This eliminates the need for users to self-report dietary intake altogether.

A Nutrition Diagnosis (Step 2) can be determined *via* sensor-based devices including the intake domain for excessive or inadequate energy and fluid intake; clinical diagnoses, such as difficulties with swallowing or chewing; and behavioral-environmental aspects, such as eating patterns and physical activity. Hand-to-mouth motions measured *via* wrist-worn devices have a moderate correlation with energy intake (18) and may be used to estimate over- and under-eating. The two eyeglasses and headband use chewing and/or swallowing motions to detect eating and irregularities in the sensor signal may potentially be developed and used to identify chewing/swallowing difficulties. All four devices are also able to detect the number of eating occasions (food consumption occasions) throughout the day as well as their clock times, revealing the patient's food consumption pattern, including whether they follow a regular eating schedule or engage in more “grazing” styles of eating. This information can help with the establishment of a nutrition diagnosis.

After a Nutrition Intervention (step 3) is agreed upon between the dietitian and the patient, the devices can be used to assist patients with adhering to the planned action(s) of the intervention. A reminder about a pre-determined goal or a strategy delivered a few seconds into a meal can guide patients' food decisions especially in settings where food is self-served throughout the meal. This is potentially feasible with the off-the-shelf glasses device as it can detect eating gestures within a very short duration of 1.1002 milliseconds from the start of the occasion. Although the devices may not be useful in settings where the user prepares or purchases their own food as the food choice would have already been made when the reminder is delivered, it may help to guide their next eating occasion.

As consultation sessions can range from weeks to months apart, sensor-based devices can enable dietitians to Monitor and Evaluate (step 4) patients' progress between sessions. By setting up web- or mobile app-based communication systems such as Healthie (83), Cronometer Pro (84), and Ascend (85), dietitians may be able to view patients' sensor-triggered dietary records in real-time and adjust the dietary intervention accordingly without delay or postponing until the next appointment. This may help to maintain patient motivation and adherence to dietary goals and plans between consultations (86).

## Discussion

Currently, traditional dietary assessment methods such as food frequency questionnaires, 24-h recalls, and food records or diaries are still the most commonly used in dietetic practice (87). However, online adaptations of these methods including web-based programs, mobile phone and smartphone applications, and other technologies have become the norm in supporting the delivery of nutrition care (88, 89). Although some authors from both dietetic and engineering backgrounds have already employed sensor-based devices into their studies (21, 24, 25), previous literature reviews in this field have not considered the real-time applicability and feasibility of sensor-based devices when used in dietetic practice settings (13, 90). This review provides an overview of the latest sensors used to detect eating occasions. The sensors were evaluated against a set of five feasibility criteria outlined in this discussion to determine whether they could feasibly be implemented into dietary assessment and potentially improve the accuracy, efficiency, and effectiveness of the NCP. This set of criteria may also be helpful for guiding engineers in the development of future devices to incorporate features that are important and practical for dietitians.

### Summary of Evidence

This scoping review identified a total of 57 devices or device sets, spanning across six categories: wrist-worn, neck-worn, ear-worn, glasses, “other” (non-wearable devices and devices worn on less common locations of the body), and a combination of devices. Of the 57, none of the devices met the feasibility criteria as suitable for dietitians to incorporate into practice settings. The most responsive device with the shortest time resolution could detect eating and/or drinking occasions within 1.1002 milliseconds from the start of an eating event, which can be used to provide timely feedback. Devices with longer time resolutions can instead be used to remind users to record their dietary intake as an automated, event-contingent EMDA.

### Feasibility Criteria for Sensors in Dietetic Practice Settings

The five criteria for feasibility of use by dietitians in real-world practice settings were: accuracy in detecting all eating episodes; evaluation of device performance in settings where participants had the freedom to select their own food and nutrition behaviors; user comfort and acceptance when worn in social settings and for long durations; detection of episodes in real-time and with little delay; and a sustainable battery life of at least one waking day on a single charge.

An average F1-score or accuracy of ≥80% in detecting eating and/or drinking was selected as the cut-off for a satisfactory performance. Only studies achieving these results in free-living, semi-free-living, or laboratory settings where participants were free to choose their own foods and activities are appropriate for real-world use. Only a small number of devices that met these two criteria had been evaluated to detect both eating and drinking occasions (62, 67, 79, 80) despite beverages contributing to up to one quarter of total daily energy intake (91, 92). This may be because beverages that are consumed continuously throughout the day such as water, tea, and coffee are difficult to detect (67) and distinguish from eating using current sensors (62, 80). Most of the devices that were able to distinguish between eating and drinking were tested on a limited number of foods and beverages (27, 37, 40, 44, 47, 49, 50, 76, 78), using differences in chewing and swallowing patterns (47, 49, 50, 78), sounds (49, 76), or wrist movements (27, 37, 44) to distinguish between the two. Additional evaluation using more food types in settings where subjects are free to choose their own foods and activities is required. The detection of beverages may also require supplementary tools such as the use of sensor-embedded drinking utensils (92) for a more accurate assessment of beverage intake.

Dietary assessments typically involve the collection of dietary data over several days and may require repeated sampling to determine nutrient intakes and food patterns. However, during dietary intervention and monitoring, food consumption may need to be measured at multiple time points over weeks, months, or even seasons to determine the amount of progress made and to continue to elicit behavior change toward nutrition-related goals (93). Therefore, it is important that the devices do not cause wearer burden (94). Few studies addressed user comfort and acceptability (34, 48, 51, 56, 60, 66, 78). Privacy concerns were raised for continuously capturing cameras (34, 67, 95) and air microphones (49). Neck devices (48, 51) and earphone-like devices (60) scored modest ratings for comfort (48, 96), whilst wrist-worn devices were readily accepted by users (78, 96). Further adjustments need to be made to the hardware of the earphone prototype to improve the fit of these devices and camera capture should only be triggered when eating is detected to address privacy concerns. For devices such as neck-worn and glasses devices, a smaller and thinner or a customizable appearance would be more socially acceptable (48, 51, 67).

Unlike traditional event-contingent EMDA, passive sensor-based devices do not require participants to initiate the self-reporting of each eating event (19), reducing the number of missed occasions resulting from when users forget or decline to report (97). However, these devices will only be effective if eating or drinking gestures are detected instantly at the start of a main meal, snack, or beverage or even predicted before the event (74). Although many papers stated that real-time data collection and processing was possible, few tested the entire data processing pipeline in real-time or calculated the anticipated time delay between eating and gesture identification (34, 45, 54, 61, 66, 67). It is important that the time delay is kept to a minimum so that dietitians are able to deliver just-in-time feedback and positively influence the user's food choice before it has been consumed. Devices should continue to be tested in real-time to ensure near instant detection or prediction before the event.

As people may eat at any time during the day, an important requirement for passive dietary monitoring is a battery life that can sustain continuous day-long monitoring. Only five papers evaluated and reported on the battery life of the device, all of which could last for at least 12 h or one waking day on a single charge either during real-time implementation or as estimated using power measurements (34, 48, 51, 54, 67). Further evaluation is required to ensure that the battery life of sensor-based devices can last or exceed the waking hours of one day even whilst operating at maximum performance.

Devices that meet the five feasibility criteria have the potential to assist in all four stages of the NCP. By passively detecting eating gestures, the sensors can be developed to remind users to record their food intake, reducing the reliance on human memory and increasing the accuracy of dietary assessment. A nutrition diagnosis can be established more efficiently *via* the pattern and frequency of food consumption as well as the estimated energy intake captured by the sensors. Dietary interventions can be made more effective when users are reminded of a predetermined goal or strategy during food intake. By remaining online, dietitians can continuously monitor and adjust the intervention without delaying until the next appointment.

### Strengths and Limitations

Our scoping review has some limitations. Studies that used discontinued device models were excluded which may have potentially omitted software and data processing pipelines that were accurate, high speed, and computationally inexpensive. In addition, there were several limitations with the data extraction process. We were not able to compare results across studies due to the lack of standardized eating or drinking outcome and evaluation measure of intake. We also only selected the “best” evaluation metric as indicated by authors to report in Supplementary Table 1. Although this was only as reported in the most realistic scenario. For example, if performance in laboratory and free-living settings were both included in the study, only the free-living performance was reported in our table. The feasibility criteria used to assess the devices' suitability for use in dietetic practice settings may also have been overly restrictive. It is possible that there were devices that were computationally inexpensive with real-time applicability but were excluded because these attributes were not evaluated in the current study identified by the scoping review. The field of wearable and non-wearable sensors is relatively young and most of the devices identified at this point in time are likely to be in preliminary stages, particularly as the review was limited to studies published in 2016 or later. They are likely to be developed further to overcome challenges such as hardware obtrusiveness in the near future.

A major strength of this scoping review is that it was written using plain language and limited technical computer science terminologies by accredited dietitians with experience in dietary assessment and MNT, making it useful for a dietetic audience. Theoreticians, nutritionists, and geospatial data scientists also contributed to the writing and development of this paper as well as the interpretation of studies identified by this review. The systematic process of the PRISMA-ScR framework used to guide this review produced replicable, transparent, and comprehensive results. The wide scope of the search (Appendix A) and inclusion of both published and unpublished literature further maximized the capture of relevant information to synthesize evidence in this emerging field.

## Conclusions

Methods of assessing food and beverage consumption that detect food consumption patterns in a more dynamic and objective manner are needed to more robustly capture a broader range of food and nutrient intakes. In this review, we identified all recently published studies describing sensor-based devices that passively detected and recorded the initiation of eating in real-time. A set of feasibility criteria based on high accuracy, user acceptability, real-world and real-time applicability, and a sustainable battery life, was applied to these studies to filter for those that were most promising for use in dietetic practice settings. Whilst most of the identified sensors and devices only embodied some of these attributes, we have demonstrated how sensor-based devices may be useful for assisting with the assessment of dietary intake as well as other stages of the NCP. As sensor-based capture of eating is a rapidly evolving field, further development, refinement, and testing of device hardware and software are likely to occur over the next few years which point toward the potential positive and tangible benefits of utilizing sensor-based devices to assess food consumption patterns.

## Author Contributions

MA-F and AR conceptualized the study. AR and LW developed the review protocol. LW conducted the literature search and wrote the first draft of the manuscript. LW and J-AY extracted the data. AR, MA-F, and JT wrote sections of the manuscript. All authors contributed to manuscript revision, read, and approved the submitted version.

## Funding

This project was funded through an International Seed Grant award awarded by the University of Sydney and the University of California San Diego (Allman-Farinelli & Hekler).

## Conflict of Interest

The authors declare that the research was conducted in the absence of any commercial or financial relationships that could be construed as a potential conflict of interest.

## Publisher's Note

All claims expressed in this article are solely those of the authors and do not necessarily represent those of their affiliated organizations, or those of the publisher, the editors and the reviewers. Any product that may be evaluated in this article, or claim that may be made by its manufacturer, is not guaranteed or endorsed by the publisher.
