# Supplementary material for: Enhancing Nutrition Care Through Real-Time, Sensor-Based Capture of Eating Occasions: A Scoping Review
Source: Front Nutr. 2022 May 2;9:852984. doi: 10.3389/fnut.2022.852984 (PMC9108538; doi:10.3389/fnut.2022.852984)
Supplement: Supplementary file 1 [file Data_Sheet_1.pdf]

frontiers

Supplementary Material

Appendix A: Search Strategy

Technology-based dietary assessment tools – MEDLINE Search Strategy (Literature Search performed: December 9, 2021)

1. Nutrition Assessment/
2. Nutrition Surveys/
3. 1 or 2
4. "Diet, Food, and Nutrition"/
5. Eating/
6. 4 or 5
7. 3 and 6
8. Eating.tw.
9. Dietary behavio?r.tw.
10. Feeding.tw.
11. "Dietary intake".tw.
12. "Food intake".tw.
13. 7 or 8 or 9 or 10 or 11 or 12
14. Monitoring, Ambulatory/
15. Wearable Electronic Devices/
16. Mobile Applications/
17. Smart Glasses/
18. Internet-Based Intervention/
19. "Ambulatory monitoring".tw.
20. "Electronic health".tw.
21. "Mobile health".tw.
22. smart?watch.tw.
23. wearable sensor*.tw.
24. wearable device*.tw.
25. "Wearable camera".tw.
26. "Ecological momentary assessment".tw.
27. 14 or 15 or 16 or 17 or 18 or 19 or 20 or 21 or 22 or 23 or 24 or 25 or 26
28. Time Factors/
29. ("early" or "earlier" or late* or hour*).tw.
30. (time or timing or schedul* or pattern* or variability or variation or detect* or identif* or recogni* or chang*).tw.
31. 28 or 29 or 30
32. 13 and 27 and 31

frontiers

Supplementary Table 1. Methods and performance of sensor-based devices that detect food and/or beverage intake (n = 54)

Ref	Sensors[a]	Intake type[b]	Ground truth/Results[c]	Advantages[d]	Disadvantages[d]	Setting[e]	Processing[f]
**Wrist-Worn Devices (n = 18)**							
27	Accelerometer (1)	Foods<br>Beverages<br><br>HTM motions	Objective ground truth: Video recording<br>Accuracy: 97.1%<br>Precision: 93.01%<br>Recall: 93.96%	Subjects are free to move/act freely around the house for an unlimited time before eating. Distinguishes between eating and drinking.	Data not collected and classified in real-time.	In-lab<br>4 foods and 1 drink<br>6 subjects	3-layer 1D CNN<br>25 Hz
28	Accelerometer (1)<br>Gyroscope (1)	Eating (not further defined)<br><br>HTM motions	Subjective ground truth: App to record and label beginning and end of each meal<br>F1: 0.82<br>Precision: 0.85<br>Recall: 0.81	Commercial Mobvoi TicWatch S smartwatch used. Able to distinguish between type of cutlery used (fork, knife, spoon, a combo of the above, or hand).	Battery life of deep learning and machine learning computations may be short. Device was trained and tested using LOSO so did not classify eating in real-time.	Free-living<br>481 hr 10 min total<br>12 subjects	CNN with HMM<br>100 Hz<br>"Small number" of features
29	Accelerometer (1)<br>Gyroscope (1)	Eating (not further defined)	Subjective: Labeled eating on audio recordings	Android smartwatch used. Able to collect data continuously for 15 hours.	Device was not tested and retraining was not performed in real-time.	Free-living<br>2 weeks<br>6/12 subjects	Deep learning<br>20 Hz<br>10 statistical features

		HTM motions	Accuracy: 0.81 to 0.89 (after retraining)	Improves accuracy with use via retraining (user provides label for critical samples) and environmental BLE.		had usable data	
30	Accelerometer (1)<br>Gyroscope (1)	Main meals<br><br>HTM motions	Objective:<br>Video recording<br>Per-meal average true +ve rate and +ve predictive value (precision): 74.5% and 95.8%	Shimmer 3 used. A range of utensils used - chopsticks, fork, hand, and spoon.	No behaviors similar to eating-related movements were tested.	In-lab<br>10 meals/ foods<br>2 subjects	Threshold value-based algorithm
31	Accelerometer (1)<br>Gyroscope (1)	Meals<br>Snacks<br><br>HTM motions	Subjective:<br>Mark start/end of each meal<br>Accuracy per person: 48% to 93%<br>Median 76%	Shimmer 3 used. 94% participants reported accuracy above 70%.	Device was not tested in real-time. Only used 60% subjects' data as 10% did not follow protocol and 30% took devices off.	Free-living<br>1 day<br>104 subjects	Naive Bayes<br>4 features
32	Accelerometer (1)<br>Gyroscope (1)	Eating (not further defined)	Subjective:<br>Mark start/end of each meal<br>Precision: 0.901<br>Recall: 0.887	Commercial smartwatch used. Device worn on hand usually operating fork or	Device was trained and tested using LOSO so did not classify eating in real-time. High sampling	Free-living<br>16 x 5 hours	CNN then LSTM<br>100 Hz<br><br>Compared with DBSCAN

		HTM motions	Specificity: 0.992<br>F1: 0.894<br>Average Jaccard Index: 0.804	spoon as opposed to dominant hand.	rate and average recording was 5 hours.	recordings<br>6 subjects	
33	Accelerometer (2)<br>Gyroscope (2)<br><br>Pandlets smartwatch worn on both wrists	Main meals (> 5 mins)<br>Beverages (pick up cup)<br><br>HTM motions	Both subj/obj:<br>1 meal: manual log of activities for 2 young subjects, 3rd person annotation for 2 senior subjects<br>1 day: manual log of activities with start/end times of eat/ drink (sec precision)<br>Precision, recall, F1 for eat: 0.39, 0.77, 0.52<br>Drink: 0.37, 0.62, 0.46	Classifier is trained first using training datasets then tested in a validation set. Results are for when a smartwatch is worn on the dominant wrist rather than both wrists. Distinguishes between eating and drinking.	Beverages require picking up motion which may not be present when drinking from straw. Activities like biting nails and playing cards classified as drinking and eating respectively. Does not recognize every single time a user eats something e.g., taking a piece of chocolate.	Validation set:<br>Free-living<br>5 subjects<br>1 meal (4 subjects) or 1 day (1 subject)	Random Forest<br>33 features<br>100 Hz<br><br>Compared with kNN, NB, DT, MLP, HMM
34	Accelerometer (1)<br>Gyroscope (1)<br>Camera (1)	Meals (at least 3 HTM in 60 sec)<br><br>HTM motions	Subjective:<br>Validate images uploaded to Annapurna Web Journal to	When eating is detected, a pop up appears on smartwatch to confirm eating. Image filter is a	Sensors turned off for 10 mins at the end of each meal - may miss 2nd serves. Sensors turned off for 3 mins during certain	Free-living<br>4-6 days (1-2 meals per day)	Decision tree for sensors<br>CNN for images<br>36 features (6 features x 3 axis x 2)

			capture false +ve<br>Precision: 63% without image filtering (sensors only), 95% with image filtering	light pre-processor on a smartphone then detailed processing on a server with CNN. Detects food intake with 60 sec delay (tested). Battery life covers waking hours of 1 day. Captures food type although does not provide assessment of food type.	activities, e.g., typing on keyboard - may miss eating at desk. Camera positioned to capture plate when hand near mouth - misses foods that are never on plate. Not able to capture false -ve and therefore no recall. Camera privacy issues.	9 subjects	
35	Accelerometer (2)<br>Gyroscope (2)	Main meals<br><br>HTM motions	Objective: Video recording<br>User-dependent<br>Precision: 0.859<br>Recall: 0.858<br>F-measure: 0.857	Other face-oriented activities performed between eating.	Microsoft Band 2 was worn on both wrists - no mention of analyzing on dominant hand only.	In-lab<br>2 meals<br>15 subjects	AdaBoost<br>6 features<br>31 Hz<br><br>Compared with RF, LinearSVC, LogisticReg, GaussianNB, DT
36	Accelerometer (1)	Meals<br>Snacks<br><br>HTM motions	No ground truth mentioned<br>Accuracy: 95.7% using wrist sensor,		Limited activities and foods. Device taped to wrist. Does not detect drinking when using binary classification.	In-lab<br>3 foods<br>1 subject	kNN (k = 9)<br>9 features<br>50.1 Hz

			binary classification, and accelerometer only				Compared with SVM, NB, RF, DT, neural network, LR
37	Accelerometer (1)<br>Gyroscope (1)	Eating<br>Drinking<br><br>HTM motions	Objective: Camcorder for home<br>GoPro for outside<br>Average accuracy 91.8% when distinguishing HTM from non-HTM customized to user	Model can be implemented in commercial devices, e.g., Fitbit or Apple Watch.	Training and test suites run offline. Subjects performed common confounding actions and specific eating behaviors for the training and test sets so not natural free-living behaviors.	Free-living but following protocol<br>Several hours<br>3 subjects	Naive Bayes<br>5 features<br>10 Hz
38	Accelerometer (1)<br>Gyroscope (1)	Meals<br>Snacks<br><br>HTM motions	Objective: Video recording<br>Motif recall: 0.88<br>Classification F-score: 0.75<br>Classification precision: 0.89<br>Feed gesture (FG) prediction: 36.5	Tested range of utensils and confounding activities.	Only showed results of 4/10 subjects in structured and 5/10 in unstructured experiment due to non-usable data. Device worn on dominant hand, but some users eat with non-dominant hand.	In-lab<br>16 actions / foods x 2 mins each<br>10 subjects	Random Forest<br>31 Hz

			FG accuracy: 0.84				
39	Accelerometer (1)<br>Acoustic (1)	Eating (not further defined)<br><br>HTM motions	Subjective: Select activity label (one of 5) on app before performing<br>Average Precision: 91.42<br>Recall: 91.53<br>F-score: 91.38 for classifying 5 activities	Separate training and testing data.	Device worn on the left wrist which is either on table or holding bowl in test set during eating activity. Type of food eaten not described. Not tested in natural free-living behavior - 5 activities performed for 10 mins each.	Test set: Free-living but following protocol<br>50 mins<br>3 subjects	Decision Tree<br>25 Hz for accelerometer<br>44.1 Hz for acoustic<br><br>Compared with RF, Bayesian Network, SVM
40	Accelerometer (1)<br>Gyroscope (1)<br>Magnetometer (1)	Eating<br>Drinking<br><br>HTM motions	No ground truth mentioned<br>98% classification accuracy for eating and drinking	Compares 5 face-centric activities. Distinguishes between eating and drinking.	Smartphones mounted on the wrist rather than a wrist device. Limited activities (drink, eat, smoke, rub eyes, call) and foods not specified.	In-lab<br>12 mins<br>4 subjects	SVM<br>20 feature vectors<br>50 Hz<br><br>Compared with kNN, DT
41	Accelerometer (1)<br>Gyroscope (1)<br>EMG (1)	Main meals<br><br>HTM motions	Objective: Video recording<br>Eating action identification<br>Precision: 0.93<br>Recall: 0.89<br>F1: 0.92	Range of foods brought from home or restaurant. Can capture eating speed by calculating time-stamp diff between each	Only tested on fork and spoon. Did not perform other behaviors that are commonly falsely detected as eating.	In-lab<br>15 mins<br>36 subjects	DNN<br>64 nodes<br>50 Hz

				cycle of picking, carrying, and putting in mouth. Plug-n-play and does not need initialization from the user.			
42	Gyroscope (1)	Eating (not further defined)<br><br>HTM motions	Subjective: Activities logged on app as sleep, eat, exercise, or other<br>Activity classification accuracy: 63.7%<br>Eating precision: 47.8%<br>Eating recall: 76.8%	Commercial smartwatch used.	Significant confusion between eating and other activities.	Free-living<br>1 month<br>1 subject	RNN and LSTM
43	Accelerometer (1)	6 food types<br><br>HTM motions	Subjective: Mark start/end of each meal<br>Average accuracy: 71.9%	Smartwatch used. Different types of utensils used – hand, fork, chopsticks, and spoon. Able to distinguish between 6 food types (ramen, pasta, bread,	Battery life of smartwatch does not last for the entire waking day and accelerometer data was only measured during mealtimes. Limited number of foods and users had to follow specific	In-lab<br>1-2 meals<br>5 subjects	Cubic SVM<br>12 features

				onigiri, gudon, cake).	instructions on how to eat.		
44	Accelerometer (1)<br>Gyroscope (1)	4 food types<br>Drinking from cup<br><br>HTM motions	No ground truth mentioned<br>Average accuracy: 97.82%<br>Average F1-score: 99.12%	Smartwatch used.	Data processing not performed in real-time but rather post hoc as study used public dataset. Transitions between activities were not continuously recorded but separately collected. Use of deep learning may result in short battery life.	In-lab<br>18 activities x 3 mins each<br>51 subjects	RNN with LSTM<br>20 Hz
**Neck-Worn Devices (n = 9)**							
45	Contact microphone (1)	Meals<br>Snacks<br><br>Eating sounds	Objective: Video recording<br>86.1% accuracy between eating and non-eating	Training occurred in a setting with ambient noise and subjects were free to do other activities. Prototype tested in real-time on attendees eating snacks during the demo. Loose-fitting device.	Types of foods trained/tested on not specified. Results of the demo are not reported. Chewing or swallowing sounds not specified. Device not specifically trained for occasions where users eat whilst performing physical activity, e.g., eating whilst walking.	Training:<br>In-lab<br>3 meals<br>2 subjects	DNN with single layer<br>23 features<br>500 Hz

Commented [LW1]: gyudon

46	Contact microphone (1)	6 foods<br>Water<br><br>Chewing<br>Swallowing	No ground truth mentioned<br>Average eating / drinking event detection accuracy: 86.6%<br>Accuracy classifying solids: 99.7%<br>Liquid: 97.6%	Detects both chewing and swallowing sounds so able to detect both liquids and solids.<br>Connected to an app which displays time of detected food, chewing pace, time since last drink, time since last meal, no. of snacks.<br>Differentiates between 7 food types, including water.	Lower accuracy for subjects with smaller BMI. Subjects required to eat food in single pieces, which is unrealistic.<br>Experiments conducted in low noise settings and subjects reduced head movement, speaking, coughing, etc. Peanut lowest accuracy (75.5%).	In-lab<br>7 food types<br>12 subjects	Event detection: HMM<br>Food type: Decision Tree<br>34 features
47	Piezoelectric (1)	16 foods<br>Liquids<br><br>Chewing<br>Swallowing	Subjective: Select food (one of 17) on smartphone app and press push button for each swallow<br>Intake detection accuracy: 89.2%	Spontaneous swallows are smaller in magnitude than food swallows so able to differentiate.<br>Distinguishes between 17 food types, including liquids, using	Subjects directed to eat one food at a time during a meal, which is unrealistic and not possible for mixed dishes. Liquids and apples have the highest error of missed swallows (but liquids had highest food type classification	In-lab<br>3 meals<br>20 subjects	Random Forest<br>15 features<br>20 Hz<br><br>Compared with kNN, SVM

			F-measure for classifying food type: 80.3%	chewing and swallowing patterns. Connected to an app which displays food type eaten, detected swallows, and when the next meal should be.	accuracy). Soft solid foods obtained lowest food type classification accuracy.		
48	Proximity (1)	Meals<br>Snacks<br><br>Chewing	Subjective: Annotate beginning and end of eating activities on Android<br>Eating episode detection<br>Precision: 78.2%<br>Recall: 72.5%	The device can differentiate between talking and chewing during in-lab. May be able to measure meal speed via individual chews before being clustered into chewing bouts. Small and discrete. Avg comfort score: 3.6/5. 86.7% of users were aware of the device during study. 73.3% wouldn't mind attending	67% F1 score for soft foods from controlled field study (cf. 73% for non-soft food). Drinking gave rise to significant sensor distance reading as the head tilts back but differs between people and occasions. Sensor common to move out of place during sports in-field.	Test set: Free-living<br>2-8 hrs (avg 4 hrs 38 min)<br>17/19 subjects included in analysis	Level-crossing<br>20 Hz

				social events with the device.<br>Battery life >18 hours.			
49	Air microphone (1)	Meals<br>Snacks<br>Beverages<br><br>Swallowing	Subjective:<br>Push button for each swallow<br>(1) F-measure: 91.3%<br>Precision & recall:<br>Sandwich: 86.3%, 88%<br>Chips: 100%, 100%<br>Water: 87.7%, 86%<br>(2) F-measure: 88.5%<br>Precision & recall:<br>Nuts: 90.6%, 78%<br>Choc: 87.5%, 98%<br>Patty: 88.2%, 90%	More comfortable as it rests loosely around the throat near the collarbone.<br>Higher accuracy than piezoelectric, especially for dry foods.<br>Distinguishes between eating and drinking and also 3 food types.	Conducted in in-lab environments with faint background noises - may not be applicable for loud free-living environments. Able to classify small amounts of foods but may not scale well to more foods (i.e., 50-100). Privacy concerns with continuous audio recording. Power overhead 10x greater than piezoelectric (44,000 Hz).	(1) In-lab, 2 food types, 1 drink type, 20 subjects<br>(2) In-lab, 3 foods, 20 subjects	Random Forest<br>4 features<br>44 kHz
49	Piezoelectric (1)	Meals<br>Snacks<br>Beverages	Both subj/obj:<br>(1) Observation	Not affected by environmental noises. Able to	Uncomfortable as requires contact with	(1) In-lab, 2 foods, 1	Random Forest<br>360 features per spectrogram swallow

		Swallowing	F-measure 75.3%<br>Sandwich: 71.1%, 74%<br>Chips: 68.7%, 66%<br>Water: 86%, 86%<br>(2) Push button for each swallow F-measure 79.4%<br>Nuts: 83.3%, 70%<br>Choc: 72.7%, 80%<br>Patty: 83%, 88%	distinguish between solids/liquids: chewing between swallow = solid, continuous swallows = liquid. Also distinguishes between 3 food types.	lower trachea during eating.	drink, 10 subjects<br>(2) In-lab, 3 foods, 20 subjects	20 Hz
50	Doppler ultrasound (2)	6 foods<br>Water<br><br>Chewing<br>Swallowing	Subjective:<br>Chew: Press buzzer whenever teeth were pressed together<br>Max accuracy: 91.4% (when no. of hidden layers, hidden nodes, length of features were 2, 10, 5)	Able to distinguish chewing jaw movements from talking.<br>Classifying water from swallow from solid foods had relatively high accuracy (86.5%). Able to distinguish between 7 food	Sensor came into contact with jaw when subject nodded or opened mouth widely.<br>Classifying apples from other foods (nugget, cracker, peanut, pizza, walnut, water) had lowest accuracy (46.7%).<br>Low accuracy for people with dysphagia related to stroke	In-lab<br>4 x 6 sub-sessions<br>10 subjects	Artificial neural network<br>4 features per frame<br>40 kHz

			Swallow: Press buzzer just prior to swallowing<br>Max accuracy: 78.4%	types, including water.	complications due to coughing and choking.		
51	IMU (1)<br>Proximity (1)<br>Ambient light (1)	Meals<br>Snacks<br><br>Chewing	Objective: Visually and acoustically from video recording<br>Per eating episode<br>Avg F1-score: 77.1%<br>Avg precision: 86.6%<br>Avg recall: 78.3%	Slight shift in necklace positioning does not affect chewing detection. 15.8-hour battery life on a single charge. Survey suggested most users were comfortable but would prefer a smaller size.	Lying down while eating reduced ability to capture LFA and chewing from the proximity sensor; eating in darkness caused ambient light to give low readings; eating during exercise caused false +ve. Non-chewing foods are hard to detect. Performance is lower when tested on subjects with obesity.	Free-living<br>2 days<br>10 subjects	Gradient boosting + DBSCAN to cluster 257 features for every chew sequence<br>20 Hz
52	IMU (1)<br>Piezoelectric (2)	Foods<br>Beverages<br><br>Swallowing	Objective: Video recording<br>Avg F-score for detecting swallows: 76.07% (lowest 52.51%)	Able to detect individual swallows with RMSE 3.34 so potentially able to differentiate beverages (continuous swallows)/solids (swallows with	Spontaneous swallows not discussed - drinking coffee at a cafe with long intervals in between may be identified as spontaneous swallows.	In-lab<br>9 actions x 2 mins each<br>10 subjects	RNN with LSTM (dimension = 32)<br>100 Hz<br><br>Compared with 64 dimension and RF

				chews in between).				
**Ear-Worn Devices (n = 9)**								
53	Contact microphone (1)	Meal<br><br>Chewing Swallowing	Objective: Video recording Precision, recall, F1:<br>Chew: 0.98, 0.91, 0.95<br>Swallow: 0.96, 1, 0.98<br>Talk: 0.96, 0.99, 0.97	Experiment conducted in different noisy environments, e.g., dining room, with family members, uni cafeteria with friends. May be developed to distinguish solids/beverages from chews and swallows.	Classification of chewing, swallowing, and talking sounds not conducted in real-time. Device only worn during mealtime even though in free-living settings. Types of food consumed not specified.	Free-living<br>1 meal (except 1 subject who had 5 meals)<br>6 subjects	Medium Gaussian SVM<br>30 features<br>8 kHz<br><br>Compared with DT, kNN, Ensemble Classifiers
54	Contact microphone (1)	Food<br><br>Chewing	Objective: Video recording<br>Accuracy: 92.8%<br>F1-score: 77.5%	Experiments ran offline but were able to capture, process, classify sensor data in real-time in free-living with mean and s.d. of delay and duration difference of 3.0 ± 3.8 minutes and 5.3 ± 5.9 minutes	Chewing is a proxy so may output false positives for chewing gum and false negatives for soft foods (yoghurt) and eating whilst walking. Unable to detect beverages. 3 min delay may miss snacks.	Free-living<br>2 hours<br>12/14 subjects had usable data	Logistic Regression<br>40 features<br>500 Hz<br><br>Compared with kNN, RF, DT, Gradient Boosting

				(calculated). Estimated 28.1-hour battery life including data-processing pipeline on a single charge. Latest design in the form of a headband is less obtrusive.			
55	Contact microphone (1)	Meal<br><br>Chewing	No ground truth mentioned<br>Accuracy: 91.5%<br>Precision: 95.1%<br>Recall: 87.4%	Actions performed during the test set included talking, coughing, and laughing. Trained on hard and soft foods including yoghurt.	Chewing is a proxy so may output false positives for chewing gum. Unable to detect beverages.	Test set:<br>In-lab<br>8 actions<br>1 subject	Logistic Regression<br>8 features<br>20 kHz
56	Accelerometer (1)<br>Gyroscope (1)	Eating (not further defined)<br><br>Head & mouth motions including swallowing	Subjective:<br>App to record beginning and end of each activity<br>Accuracy for classifying 7 activities: 93.76%	Small and discrete device.	Speaking confused with eating due to mouth movement and eating confused with stay due to sedentary. Types of food consumed are not specified.	In-lab<br>7 actions x 3 mins<br>No. of subjects not specified	CNN<br>50 Hz<br><br>Compared with RF, linear discriminant analysis, SVM, KNN

57	Proximity (1)	Food<br><br>Chewing	Subjective:<br>Manual counter to count individual chews<br>Precision: ≥ 0.958<br>Recall: ≥ 0.937	Counts individual chews so can determine eating speed. Displays chewing info on tablet in real time. Small and discrete earphone-like device.	Amplification is adjusted manually by researchers before each food so chewing event timeframe is controlled and monitored for data collection. Swallowing saliva and using tongue to remove stuck food were recognized as chewing; chewing was missed when subjects used less force. May not be applicable for softer foods as only chewing gum and almonds used in this experiment.	In-lab<br>2 foods (300 chews each)<br>6 subjects	Threshold value-based algorithm<br>250 Hz
58	Proximity (1) (worn in 1 ear)	Food (meals)<br><br>Chewing	Both subj/obj:<br>Wore PC to record voltages and times of voltages from sensor + questionnaire about meal start/end times<br>Mealtimes estimated from	Small and discrete earphone-like device. Can be worn in just 1 ear.	Mealtime estimates occur in 5 min intervals rather than exact minute/second. Sensor wearing time (e.g., 2 hr) decided before detecting meal start/end time so not able to detect without prior user input. Algorithm based on	Free-living<br>2 hours<br>7 subjects	10 Hz

			changes in ear canal within 5 mins of ground truth		assumption that wearing time will always include mealtimes.		
	Proximity (2) (worn in both ears)	Food (gum)<br><br>Chewing	Objective: Wore PC to record voltages and times of voltages from sensor<br>Pearson's product-moment correlation coefficient of L and R ear in running ≥ 0.741 but ≤ 0.384 in chewing	Small and discrete earphone-like device	Only comparing chewing of gum and running - may not work for comparing, e.g., eating yoghurt with talking. Worn in both ears as diff between chewing gum and running is that chewing gum has low correlation coefficient between ears and running has high.	In-lab<br>Chewing gum and running<br>4 subjects	10 Hz
59	EMG (1)	Main meals<br>Snacks (> 5 sec)<br><br>Chewing	Subjective: Marked start/end of eating on recording device + duration and type (meal/snack) on app<br>After exclusion of 2 participants (<5 hrs recording):	2 unobtrusive electrodes positioned behind the ear rather than on the face or neck.	Detected chewing phases < 5 sec discarded; chewing phases < 15 sec apart merged; merged phases < 20 sec discarded. Without including bruxism / nail biting subjects, mean sensitivity and specificity were 90.6% and 92.1% so they can	Free-living<br>20 hours<br>15 subjects	512 Hz

			Sensitivity: 90.6%<br>Specificity: 92.1%		lower results. Strong head movements triggered false +ve.		
60	PPG (1)<br>Air microphone (1)<br>Accelerometer (1)	Main meals<br>Snacks<br><br>Chewing	Objective: Researchers monitored subjects and created diaries + marked start/end of eating on audio files<br>LOSO duration-based evaluation<br>Precision: 0.794<br>Recall: 0.807<br>Accuracy: 0.938<br>Class-weighted accuracy: 0.892<br>F1-score: 0.761	Apart from eating lunch and dinner at uni, subjects could spend the afternoon freely but must eat 3 snacks and perform 4 activities. Signal from accelerometer helps to differentiate physical activity and eating. Soft foods such as ice cream, puree, and custard were consumed.	Testing not conducted in real-time so unable to comment on battery life. Earphone with ear hook-like device is currently one-size-fits-all which may result in incorrect placement. Uses chewing as a proxy so may not be able to detect hard candy or soups. Subjects ranked comfort of chewing sensor 3.8/10, able to wear for 3.9 hrs per day, 19/20 scored more than 5/9 for sensor bothers them, they found it painful, cable annoying, reduced hearing	Semi-free living<br>1 or 2 days<br>14/22 subjects had usable data	SVM<br>PPG: 10 features, 21.3 Hz<br>Microphone: 15 features, 48 kHz<br>Accelerometer: 21.3 Hz
61	IMU (2)<br>Microphone (1)	Meals<br>Snacks<br><br>Chewing	Objective: Video recording<br>Chewing<br>Accuracy: 93%	Eating episodes ranged from 2 min snacks to 30 min meals. Average	Did not recognize frozen yoghurt and classified talking as eating. Short battery	Test set: Free-living	Random Forest<br>34 features<br>50 Hz

			F1-score: 80.1%<br>Precision: 81.2%<br>Recall: 79%<br>Eating episode 15/16 recognized<br>2 false positives	delay is 65.4 sec (calculated rather than tested in real-time).	life of ground truth cameras limited free-living sessions to 3 hours. All components of the device need to be in a precise position. Testing not conducted in real-time.	2 x 3-hour sessions<br>10 subjects	
**Glasses Devices (n = 7)**							
62	Accelerometer (1)	Eating events (including liquids)<br><br>Chewing	Subjective: Subjects kept log of all eating events (solids and liquids)<br>F1-score for 20 s epoch: 85.8% +/- 11.7%	Both meal (pizza, pasta, sandwiches, fried rice, salads) and snack (fruit, nuts) foods were tested. Able to detect both food and beverage intake.	Unable to differentiate between foods and liquids. Eating while walking or during other PA was not trained or explicitly tested. Data processed offline for algorithm development and evaluation.	Test set: Free-living<br>3 hours<br>8 subjects	kNN (k = 10)<br>3-12 features per fold<br>100 Hz
63	Piezoelectric (1)	2 foods (pizza, granola bar)<br><br>Chewing	Subjective: Push button to mark start/end of each activity<br>Average F1-score of classifying 4 activities (sedentary, eat +	Decision tree is quick and appropriate for real-time classification.	Online real-time classification of recorded behaviors from 3 subjects rather than real-time behaviors. Eating + sitting misclassified as sedentary and eating +	Test set: In-lab<br>5 actions (rest, eat + sit, talk, eat + walk, walk)	Decision Tree<br>5 features

			sit, eat + walk, walk): 94.69%		walking misclassified as walking.	3 subjects	
64	EMG (2)	Eating (not further defined)<br><br>Chewing	Subjective: Manually log every eating event to 1 min resolution<br>20 s overlapping sliding window<br>F1-score: 95.2%<br>Starting timing error: 24.8 +/- 29.8 s<br>End timing error: 24.6 +/- 50.9 s	Ground truth method is burdensome and may be unreliable. Start timing error 24.8 s so may be able to use just-in-time interventions.	Measures temporalis muscle activation which may not be present for soft foods and soups and present for chewing gum. Types of foods consumed not specified. Device was trained and tested using LOPO so did not classify eating in real-time.	Free-living<br>1 day<br>10 subjects	One-class SVM<br>6 features<br>256 Hz
65	Load cell (2)	3 foods (bread, chips, jellybeans)<br><br>Chewing	No ground truth mentioned<br>Average F1 score in classifying 6 actions: 94%	Sensor will not be affected by perspiration or hairs between skin and glasses. Chewing was able to be differentiated from talking. Experimental amplification factor at hinge almost the same	Glasses must be tightly fitted and touching temples for force to be > 0.5 N and amplified at hinge. Foods that require minimal chewing (yoghurt, ice cream, etc.) may exert force < 0.5 N. Not tested in free-living so unsure of how the device would perform in	In-lab<br>6 actions (talk, head movement, L & R chew, L & R wink)<br>10 subjects	RBF-SVM<br>84 features

				as theoretical value.	presence of physical activity.		
66	Accelerometer (1)<br>Camera (1)	Main meals and snacks<br><br>Chewing<br>Head motions	Objective:<br>Manual image review<br>Accuracy 97.62%	Calculated time requirement for feature computation and classification: 1.1002 ms. Sensors can be attached to users' own glasses.	Low F1 during training due to non-food epochs detected as food intake. F1 of free-living not mentioned. Privacy concerns with cameras are reduced if images are only taken during meals. Not tested in real-time.	Test set: Free-living<br>8 days total<br>4 subjects	Decision tree<br>4 features
67	Gyroscope (5)<br>Accelerometer (1)<br>Proximity (1)<br>Camera (1)	Eating (> 3 sec)<br>Drinking<br><br>Chewing<br>Swallowing<br>HTM motions	Objective:<br>Video recording<br>Intake level (bite then > 3 sec chew or swallow)<br>Recall: 75.4%<br>Precision: 84.7%<br>Coverage: 68.2%<br>Episode level (intake within 5 min combined)<br>Detected 22/28<br>Coverage: 89%<br>4 false +ve	FitByte can run for 16.5 hours using onboard battery. Able to detect drinking where duration between sips < 30 sec but not distinguish from eating. 6.5 sec delay (tested) in detecting eating/drinking episodes. Participants reported not feeling	Not able to detect drinking when sporadic, short sips mixed with other noisy activities (e.g., hiking, reading book in cafe). Participants would prefer if glasses frames could be customizable and had privacy concerns with the onboard camera being on the entire time.	Test set: Free-living<br>12 hours<br>5 subjects	Random Forest<br>Gyroscope: 156 features, 50 Hz<br>Proximity: 6 features, 50 Hz<br>Accelerometer: 6 features per bin, 4 kHz

				uncomfortable wearing glasses. Captures food type but does not provide assessment.			
68	Proximity (1)	Chewing	Subjective: 2 push buttons - one for eating, one for individual chews<br>20: Accuracy 97.6%, F1 97.6%<br>21: Accuracy 97.3%	In 20, chewing count and chewing rate extracted, so can determine speed of eating.	Limited foods (3 spoons of 3 types of foods) and activities (rest only). Only 1 subject.	In-lab<br>3 foods<br>1 subject	SVM<br>40 features<br>50 Hz<br><br>Compared with Ensemble: Boosted Tree
**Other Devices (n = 10)**							
69	Transmitter (1)<br>Receiver (1)<br><br>WiFi	Eating<br><br>HTM motions	Objective: Camera based method for eating duration<br>Eating gesture accuracy: 97.8%<br>Accuracy in classifying, duration error<br>Spoon: 90%, 23 s<br>Fork: 94%, 18 s	Can measure time, duration, and speed of eating by deriving start/end of eating. Tested on a range of utensils. Imitates scenarios when users are eating and placing smartphones on the dining table.	Requires precise placement of WiFi access point and smartphone for WiFi signal to detect eating. WiFi access points are not always present during eating occasions, especially snacks or eating on the go. Tested in-lab settings and non-	In-lab<br>400 min total<br>10 subjects	Discriminant Analysis Classifier

			Fork/knife: 100%, 14 s<br>Hand: 97%, 44 s<br>Chopsticks: 100%, 16 s		eating behaviors not performed.		
70	Contact microphone (1) located on tip of mastoid bone in headband	Chewing via jaw movement	No ground truth mentioned<br>Accuracy: 95.15%<br>F1: 94.89%<br>Precision 94.68%<br>Recall 95.12%.	6 ms latency in classifying eating/non-eating.	Eating and non-eating activities were performed separately. Post hoc analysis of collected data.	In-lab<br>6 eating activities, 7 non-eating activities<br>30 subjects	Shallow GRU neural network<br>20 kHz<br>STFT features
71	Accelerometer (1)<br><br>Temporalis muscle headband	Chewing	Objective: EMG for no. of chews<br>Eating activity detection<br>Accuracy: 97.1%<br>F1-score: 93.6%<br>Chew count average error rate: 12.2%	Able to detect individual chew counts so can measure eating speed. Speaking, standing, sitting, walking, drinking, coughing, and behaviors commonly detected as eating, were performed.	Watermelon was the only food trained and tested. Only one subject performed the 6 non-eating activities and cross validation was used.	In-lab<br>7 actions<br>4 subjects	Decision Tree<br>23 features<br>100 Hz<br><br>Compared with nearest neighbor, MLP, SVM, WSVM
72	Accelerometer (1)<br>Gyroscope (1)<br>Load cell (1)	Meals<br><br>Use of fork	Objective: Video recording and weight of food	Detects both gestures and weight of food on fork so could	Can only be used in foods that use a fork. Only tested on 2 foods. Bulky design.	In-lab<br>2 foods<br>12 subjects	Threshold value-based algorithm<br>100 Hz

			Gesture detection sensitivity: 89.38%<br>Mean absolute % error of weight estimation: 26.297%	potentially estimate kJ consumption.			
73	Accelerometer (1)<br>Gyroscope (1)<br><br>Chin	Eating episode<br><br>Chewing	Objective: Video recording (10 sec vid every min)<br>Eating episode (rather than chewing bout)<br>Precision: 0.923<br>Recall: 0.890<br>F1-score: 0.906	Although food types are not specified in free-living, the in-lab component of the paper tested yoghurt and ice cream.	Ground truth method may have missed quick eating episodes such as putting candy in mouth. Food types not specified; may detect gum as eating and beverages may not be detected.	Free-living<br>6 hours<br>14/15 subjects had usable data	Random Forest<br>24 features per frame<br>20 Hz
30	Accelerometer (1)<br>Gyroscope (1)<br><br>Finger sensor	Main meals<br><br>HTM motions	Objective: Video recording<br>Per-meal average true +ve rate and +ve predictive value (precision): 77.9% and 95.8%	Shimmer 3 used<br>Range of utensils used - chopsticks, fork, hand, spoon.	No behaviors similar to eating-related movements were tested, e.g., touching face.	In-lab<br>10 meals / foods<br>2 subjects	Classic algorithm using 4 wrist roll events

36	Accelerometer (1)<br>Gyroscope (1)<br><br>Finger sensor	3 foods<br><br>HTM motions	No ground truth mentioned<br>Accuracy: 97.1% using finger sensor, binary classification		Limited activities and foods: noodles with chopsticks, rice with spoon, chips with hand, drinking, using computer mouse, typing. Device taped to finger. Does not detect drinking when using binary classification.	In-lab<br>3 foods<br>1 subject	kNN (k = 9)<br>22 features<br>50.1 Hz<br><br>Compared with SVM, NB, RF, DT, neural network, LR
74	Accelerometer (1)<br>Temperature sensor (1)<br><br>Lower teeth	Food intake<br><br>Chewing	No ground truth mentioned<br>Classified 9 foods into 5 classes with 85% weighted accuracy	Able to categorize food types: dry (almonds), dry hot (chips), moist hot (steak), moist cold (fruit), cold (yoghurt).	Comfort of wearing a device on lower teeth.	In-lab<br>9 foods<br>4 subjects	Random Forest<br>24 features<br>54 Hz
			Subjective: Data manually labelled on smartphone whilst being collected<br>Eating activities detected with precision and recall of 93% and 96%	Able to differentiate eating from talking, physical activity, and sedentary activities. May be used to differentiate foods and beverages (no chew + cold or hot).	Only 2 eating events (bread and pasta) for the entire 9.5 hours. Only 1 subject.	Free-living<br>9.5 hours<br>1 subject	Random Forest<br>24 features<br>54 Hz

75	Accelerometer (1)<br>GPS (1)<br><br>Worn on hip	Eating<br>Food purchasing<br><br>User location<br>Clock time	Objective: Images taken every min on front-facing camera<br>Mean accuracy of predicting eating and food purchasing 0-4 mins before: 0.7289 and 0.7395	May apply just-in-time intervention and remind users of goals if eating or food purchasing is predicted. Applies to in-home settings as well.		Free-living<br>7 days<br>81 subjects	Gradient Boosting for eating<br>RBF-SVM for food purchasing<br><br>35 features<br><br>Compared GB, RF, RBF-SVM, LR
76	Acoustic microphone (2)<br><br>Worn on collar	4 food types<br>Drinking water<br><br>Chewing<br>Swallowing	Objective: Video recording<br>Accuracy: 98.23%	Comfortable and relatively discreet device. Can detect both chewing and swallowing via audio, differentiating between eating and drinking.	Limited number of foods and behaviors. Impact of environmental noise on chewing and swallowing sounds not tested. Soft foods were not tested.	In-lab<br>7 actions x 2-4 mins each<br>8 subjects	CNN
**Multi-Position Devices (n = 4)**							
77	Accelerometer, gyroscope, magnetometer on wrist and upper arm	Eating<br><br>Arm motions	No ground truth mentioned<br>Classification accuracy of eating, teeth brushing, shaving: 98%	Uses wrist motion so could potentially detect beverages too.	Requires streaming to the host computer to record data and detect eating. Only tested 3 activities. DCM (estimates hand position relative to	In-lab<br>25 mins<br>1 subject	SVM<br>50 Hz

					body) tested separately to SVM (eating/not eating).		
78	(1) Wrist sensor with accelerometer and gyroscope<br>(2) Piezoelectric sensor on neck	5 foods<br>Water<br><br>HTM motions<br>Swallowing	No ground truth mentioned<br>(1) 8 activities classified with 94.3% accuracy (eating highest: 99.7% recall, 99.5% precision)<br>(2) 6 foods classified with 91.9% accuracy (water highest: 98.8% recall, 99.7% precision)	Able to differentiate solid/liquid with high accuracy via piezoelectric spectrogram images and CNN. User surveys suggested they were comfortable with watch/necklaces. Able to distinguish between 6 food types, including water.	(1) Subjects did not eat and perform other activities (walk, talk) at the same time. Gestures similar to eating are not performed.<br>(2) Only 6 food types (chips, cookies, nuts, pizza, salad, water).	In-lab<br>8 actions x 3 sessions<br>20 subjects	(1) SVM for activity<br>8 features<br>(2) CNN for food type
79	Hand gesture proximity sensor<br>Piezoelectric on jaw<br>Accelerometer on neck	Food intake<br>Drink<br><br>HTM motions<br>Chewing	Objective:<br>Video recording<br>Food intake bout<br>Kappa: 0.76<br>F1-score: 0.78<br>Activity type (eat, drink, rest, walk, talk)<br>Kappa: 0.77<br>F1-score: 0.8	In-lab but conducted in an apartment where subjects are not restricted in food intake or activities. Able to detect both food and beverage intake and	3 components to this sensor which may be uncomfortable for the user. Moderate agreement among raters for video annotation: 0.74 Light’s kappa for marking food intake event boundaries.	In-lab<br>3 days<br>40 subjects	3-layer neural network<br>Proximity 10 Hz<br>Piezoelectric 1000 Hz<br>Accelerometer 100 Hz

				distinguish between the two.			
80	2 microphones in 1 earbud Accelerometer and gyroscope on 2 smartwatches	Main meals Snacks Beverages<br><br>Chewing	Subjective: participant log start/end of mealtimes Intake precision, recall, and F1: 63, 88, 74.	Commercial LG G Watch used. Beverages detected. Detects both food and beverage intake.	2 smartwatches worn - may compromise user acceptability. Classified on previously collected data. Shortest meal of eating chocolate for 10.6 sec missed. Unable to differentiate between food and beverage intake.	Free-living 11 subjects 2 x 12 hrs (10 subjects) or 5 x 12 hrs (1 subject)	SVDKL: combo of DNN and multiple Gaussian Processes 44.1 kHz for microphone, 15 Hz for smartwatch Compared with RF, LSTM, SVDKL w/o initialization

[a]The number and type(s) of sensor(s) used. If devices placed on more than one location of the body were used simultaneously in a study, it was summarized under “Multi-Position Devices”. If devices placed on separate locations of the body were compared in a study, they were tabulated separately in their respective locations.
[b]The type of intake and eating proxy measured. This includes main meals, snacks, beverages, eating episodes not further defined by authors, and individual foods and/or beverages if a limited number was used and specified in the study.
[c]Ground truth method and evaluation metric(s) of sensors. The ground truth is information that is considered real or true. It was used as a comparison against algorithm outputs to evaluate the performance of the device. It can be objective, such as a video recording of the device in use or subjective, such as user self-report via an activity log. Any evaluation metric reported either in the text, a table, or a figure, that described the performance of the sensor(s) on detecting eating and/or drinking was reported. The best performing method or algorithm result as indicated by the authors was reported for the most realistic setting, e.g., free-living.
[d]Advantages and disadvantages of the study, including the study design as well as the device’s feasibility for assisting dietitians in conducting dietary assessments in real-world practice settings. Feasibility was based on the device’s accuracy, user acceptability, real-world and real-time applicability, and battery life.
[e]Experiment details including setting, duration, and number of participants. If a paper evaluated the performance of a device in both laboratory and free-living settings, only the results of the free-living setting were presented unless the device was evaluated for different foods in different settings. For example, both settings will be included if the laboratory experiment evaluated beverage intake and the free-

living experiment evaluated snack intake. Furthermore, if a study used separate sets to train and validate a device, only the validation set was presented.
[f]The data processing pipeline including algorithm used, sampling rate, and the number of features if available. If the authors compared different algorithms, they were also included in the table. The number of papers using each type of algorithm was tabulated in a separate table (Supplementary Table 2)

Supplementary Table 2. Frequency and percentage of algorithm types used in included studies, ordered by frequency.

**Algorithm used**	**Frequency**	**% of 54 studies**	**References with devices that used this classifier**
Neural Networks<br>*(computing systems inspired by the biological neural networks that constitute animal brains; used for classification and prediction)*	18	33%	27, 32, 33, 34, 36, 41, 42, 44, 45, 50, 52, 56, 70, 71, 76, 78, 79, 80
Random Forest<br>*(used for classification (0 and 1) and regression (continuous values))*	16	30%	33, 35, 36, 38, 39, 47, 49, 52, 54, 56, 61, 67, 73, 74, 75, 80
Support Vector Machine<br>*(used for classification, regression, and outlier detection)*	16	30%	35, 36, 39, 40, 43, 47, 53, 56, 60, 64, 65, 68, 71, 75, 77, 78
Decision Tree<br>*(used for classification and regression; high speed)*	12	22%	33, 34, 35, 36, 40, 46, 53, 54, 63, 66, 71
Nearest Neighbour<br>*(used for classification and regression when supervised)*	9	17%	33, 36, 40, 47, 53, 54, 56, 62, 71
Naives Bayes<br>*(used for classification and does not work with regression)*	5	9%	31, 33, 35, 36, 37
Logistic Regression<br>*(a statistical method used for predicting binary classes; high speed)*	5	9%	35, 36, 54, 55, 75
Threshold value-based algorithm<br>(*compare signals with a set of defined threshold values)*	4	7%	30, 48, 57, 72
Gradient Boosting<br>*(used to boost the performance of machine learning models; used for classification and regression)*	3	6%	51, 54, 75
DBSCAN<br>*(data clustering algorithm)*	2	4%	32, 51

Hidden Markov Model<br>*(used to predict a sequence of unknown variables from a set of observed variables; good for temporal pattern recognition)*	2	4%	33, 46
Ensemble Classifiers<br>(*used to improve machine learning results by combining individual models*)	2	4%	53, 68
Linear Discriminant Analysis<br>*(used for multiclass classification and dimensionality reduction)*	2	4%	56, 69
Gaussian Processes<br>*(used for regression and probabilistic classification problems)*	1	2%	80
Bayesian Network<br>*(a probabilistic graphical model that represents a set of variables and their conditional dependencies)*	1	2%	39
Adaptive Boosting<br>*(used to boost the performance of machine learning models; used for classification and regression)*	1	2%	35

3
